# Editorial: Agricultural sensors and systems for field detection

**DOI:** 10.3389/fpls.2023.1266610

**Published:** 2023-09-08

**Authors:** Jun Steed Huang, Ning Yang, Jianfeng Ping

**Affiliations:** ^1^Department of Systems, Carleton University, Ottawa, ON, Canada; ^2^Department of Bioelectronics, Jiangsu University, Zhenjiang, China; ^3^Department of Biosystems, Zhejiang University, Hangzhou, China

**Keywords:** terahertz, wearable, sensor, image - based modelling, anti-interference, non-destructive

The extensive use of numerous agricultural sensors can facilitate meticulous supervision of agricultural activities. However, several obstacles are currently encountered while using these sensors in the agricultural field, such as external disturbances, model inconsistency, inefficient data transmission, and high expenses. These issues hinder their extensive use in challenging agricultural settings, such as fields and greenhouses, throughout the various production phases.

Once we find solutions to these critical technical difficulties, agricultural sensors can adjust to intricate agricultural conditions, deliver consistent and precise data, offer a more trustworthy foundation for agricultural decision-making and management, and enhance the intelligence of agricultural production cycles. Simultaneously, implementing agricultural sensors will encourage the advancement and evolution of associated technologies. This includes sensor networks, data transmission methodologies, and artificial intelligence algorithms, thereby fostering the enhancement of agricultural information technology.

In light of the context, as mentioned earlier, the articles included in this Research Topic addressed several key issues. Some studies explored the technique of harnessing multi-source and multi-scale data to obtain detailed crop information, focusing on precision perception technology. This involves developing methods to accurately sense crop features and conditions by amalgamating diverse data such as remote sensing data and ground observation sensor data. An article delved into advanced, non-invasive testing technologies with robust anti-interference capabilities and model adaptability. These technologies can precisely detect crop anomalies, such as pests, diseases, and nutritional deficiencies, while effectively resisting environmental, lighting, and weather-related disturbances. Other studies investigated efficient data processing and transmission technologies suitable for field or greenhouse conditions. This includes the development of effective data processing algorithms and transmission protocols to collect, analyze, and store vast quantities of agricultural data in real time, thereby enhancing data utilization efficiency and offering prompt and precise support for agricultural decision-making. Another study focused on developing inexpensive, specialized sensor chips and systems for field detection. This will enable agricultural workers to utilize sensor equipment that is cost-effective, easy to deploy, and use. It will enable real-time crop growth and environmental monitoring, facilitating more accurate decision-making. Finally, we also have studies (Mu et al., Zang et al., Huan et al.) that examined wearable, portable sensors for plants. In direct contact with plants, these sensors will monitor plant physiological indicators and environmental parameters in real time, providing detailed insights into the plant growth process and helping optimize agricultural management and breeding practices.

The primary goal of the aforementioned research and execution is to foster technological innovation in the agricultural sector. As sensor technologies such as terahertz and 3D time-of-flying cameras (Zhang et al.) evolve and the costs of deep learning (Yang et al., Sawyer et al., Wu et al., Wang et al.) software diminish, the widespread and large-scale implementation of agricultural sensors in challenging environments for all stages of the production cycle becomes more feasible. These agricultural sensors can offer extensive and accurate data support by facilitating real-time (Thilakarathne et al.) monitoring and precise detection of various environmental factors and crop growth conditions. Technological advancements will revolutionize traditional farming practices, steering the agricultural sector towards smart and information-driven methodologies. They will play a pivotal role in ensuring the sustainable development of the agriculture industry and securing food supply.

## Author contributions

JH: Writing – review & editing. NY: Writing – original draft. JP: Writing – review & editing.

